# Single nucleotide polymorphism-based dispersal estimates using noninvasive sampling

**DOI:** 10.1002/ece3.1588

**Published:** 2015-07-07

**Authors:** Anita J Norman, Göran Spong

**Affiliations:** Molecular Ecology Group, Department of Wildlife, Fish and Environmental Studies, Swedish University of Agricultural SciencesSE-901 83, Umeå, Sweden

**Keywords:** Carnivore, citizen science, isolation-by-distance, natal dispersal, pedigree reconstruction, SNPs, *Ursus arctos*

## Abstract

Quantifying dispersal within wild populations is an important but challenging task. Here we present a method to estimate contemporary, individual-based dispersal distance from noninvasively collected samples using a specialized panel of 96 SNPs (single nucleotide polymorphisms). One main issue in conducting dispersal studies is the requirement for a high sampling resolution at a geographic scale appropriate for capturing the majority of dispersal events. In this study, fecal samples of brown bear (*Ursus arctos)* were collected by volunteer citizens, resulting in a high sampling resolution spanning over 45,000 km^2^ in Gävleborg and Dalarna counties in Sweden. SNP genotypes were obtained for unique individuals sampled (*n* = 433) and subsequently used to reconstruct pedigrees. A Mantel test for isolation by distance suggests that the sampling scale was appropriate for females but not for males, which are known to disperse long distances. Euclidean distance was estimated between mother and offspring pairs identified through the reconstructed pedigrees. The mean dispersal distance was 12.9 km (SE 3.2) and 33.8 km (SE 6.8) for females and males, respectively. These results were significantly different (Wilcoxon’s rank-sum test: *P*-value = 0.02) and are in agreement with the previously identified pattern of male-biased dispersal. Our results illustrate the potential of using a combination of noninvasively collected samples at high resolution and specialized SNPs for pedigree-based dispersal models.

## Introduction

Knowledge of dispersal patterns in wild populations can benefit research and conservation efforts, but dispersal is notoriously difficult to study (Dieckmann et al. 1999; Nathan 2001; Trakhtenbrot et al. 2005; Driscoll et al. 2014). This is especially true for sensitive, wide-ranging, and elusive species. Several empirical methods have been used to study dispersal, including CMR (capture–mark–recapture), radio-tracking, and genetics (Nathan et al. 2003; Broquet and Petit 2009; Baguette et al. 2012). However, each method has its limitations. For example, CMR methods risk missing long-distance dispersers due to a limited sampling scope (Koenig et al. 1996) and typically require direct handling of individuals possibly affecting their behavior and even survival (Kock et al. 1987). Radio-tracking captures long-distance dispersers that other methods miss (Koenig et al. 1996) and reveals fine-scale details of movement pathways and timing of departure and arrival. However, it requires expensive and highly specialized equipment as well as the need to capture and handle individuals, making it difficult to generate a large enough sample.

Genetic methods have the advantage that samples can be obtained noninvasively (Lawson Handley and Perrin 2007) and contain information that projects beyond the sampled individual (e.g., kinship). But there are many practical issues with genetic methods including, but not limited to, sampling a large enough proportion of the population, obtaining high-quality DNA from noninvasively collected samples, unknown age of individuals, unknown directionality of PO (parent–offspring) relations, assessing whether dispersal has occurred at the time of sampling, and establishing accurate pre- and postdispersal locations. Moreover, many genetic dispersal models have been developed in a population genetics framework (Wright 1943; Waser and Strobeck 1998; Gandon and Rousset 1999; Rousset 2001), where stringent assumptions of ideal populations and results that reflect historic population averages severely limit the usefulness of such models for contemporary processes (Sugg et al. 1996; Palsbøll et al. 2013). A lack of genetic resolution has largely prevented alternative approaches for all but the most intensively studied populations, where a combination of observational and genetic inferences has allowed for the reconstruction of accurate pedigrees (e.g., Pemberton 2008; Spong et al. 2008). But if some or all of these issues could be resolved, genetic techniques can be quite effective for measuring dispersal (Nathan 2001; Baguette et al. 2012).

SNPs (Single nucleotide polymorphisms) are suitable for many types of studies as they offer high genomic resolution, reproducibility across laboratories, ease of allelic assignment, and, relative to microsatellites, a reduction in erroneous results due to mistyping and allelic dropout (Anderson and Garza 2006). However, SNPs have only recently been added to the molecular toolbox due to the recent and rapid advancement of sequencing technology. As any one SNP has low statistical power compared to a multiallelic microsatellite, many more SNPs are necessary, but with today’s technology, finding many genomewide SNPs is no harder than finding a few. Choice of molecular marker should be weighed according to the biological question being asked as they afford different properties. SNPs are useful for identifying individuals and inferring relatedness given the right characteristics (Glover et al. 2010). For example, SNPs that have high minor allele frequencies, where both alleles are common within the population of interest and which are unlinked to all other SNPs, tend to be most informative for individual identification and relatedness inference (Anderson and Garza 2006).

One approach to estimating individual-based dispersal distances using molecular markers is through inference of relatedness between individuals (e.g., Spong and Creel 2001; Rollins et al. 2012), in particular mother–offspring pairs. With knowledge of individual locations, measuring the geographic distance between mother and offspring will give an estimate of dispersal distance. However, identifying mother–offspring pairs with molecular markers alone is not sufficient due to the uncertainty of directionality (i.e., it is not directly apparent which individual is the parent and which is the offspring when the relationship is assessed using molecular markers and in the absence of demographic data). As this is an essential component for estimating natal dispersal distance, at least for dyads that include a male, one must take it a step further. One way to resolve this is to attempt to reconstruct the pedigree and thus reveal the directionality of the relationship. To do this, it is critical to obtain enough samples as the higher the proportion of individuals sampled, the more complete the pedigree will be (Pemberton 2008). With a high sampling resolution, the possibility of identifying PO triads (i.e., both parents and offspring) becomes greater. These triads provide higher confidence in determining directionality through allele sharing alone as offspring will share at least one allele that is identical by descent with both mother and father at every locus.

**Figure 1:**
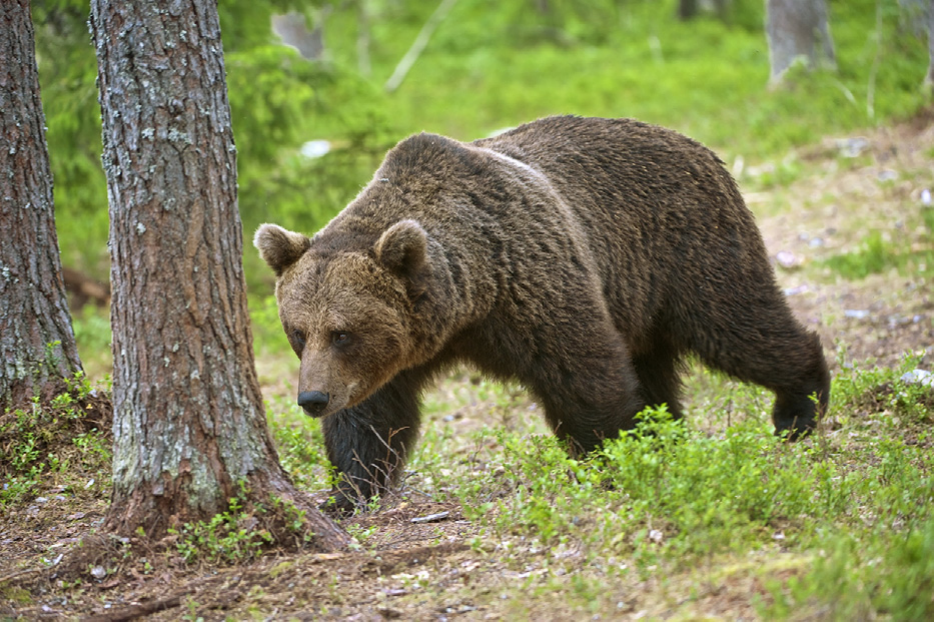
Brown bear in motion. (Photo: TT Nyhetsbyrån)

The requirement for a large proportion of samples from the population of interest can make sampling effort both time- and cost-intensive, not to mention logistically challenging. However, with a combination of noninvasive sampling and citizen participation, it is possible to achieve a high sampling resolution in a timely, cost-effective way that can be made logistically feasible. Noninvasive sampling is concentrated around locating sources of DNA, which can be found in feces, fur, feathers, saliva, and urine among others eliminating the need to interact with the study subjects (Taberlet and Luikart 1999; Taberlet et al. 1999; Waits and Paetkau 2005). Engaging local citizens who are willing to volunteer to collect samples can be advantageous as it considerably reduces costs and collection time when there are many participants. (Bonney et al. 2009; Devictor et al. 2010; Dickinson et al. 2010). An added benefit is that citizens can be knowledgeable about locating and identifying samples, thereby enhancing collection success. For over a decade, Sweden has successfully engaged citizen volunteers to help collect samples on multiple occasions from feces left by the brown bear (*Ursus arctos;* Fig.1) (see Bellemain et al. 2005). Resampling has enabled monitoring of the same population over time and has revealed population growth and declines in certain counties within Sweden (Kindberg et al. 2011). Moreover, the data generated from these collections are useful for many other applications such as identifying and tracking individuals (Kindberg et al. 2011), assessing gene flow patterns, and detecting population substructuring (Schregel et al. 2012; Kopatz et al. 2014).

Here we use a recently developed SNP panel containing 96 SNPs derived from the Scandinavian brown bear (see Norman et al. 2013) to estimate dispersal distance in the Swedish south-central population of brown bear. The SNP panel was developed for inferring relatedness between individuals, making it suitable for estimating individual-based (direct) dispersal. This study uses SNP genotyping on noninvasive samples collected by citizens to estimate dispersal distances through pedigree reconstruction.

## Materials and Methods

### Sample collection

Samples of brown bear feces were collected in a twelve-week period between August and October 2012 in the counties of Dalarna and Gävleborg, which consists of the majority of the south-central Swedish population representing the western European lineage (Taberlet and Bouvet 1994). Volunteers, mainly moose hunters, opportunistically collected feces and sent the samples along with the coordinates of the sample location to the county administration board (Länstyrelsen, Sweden). This sample collection was performed following the same protocol described in Bellemain et al. (2005) and Kindberg et al. (2011).

### DNA extraction and SNP genotyping

Samples were sent to Bioforsk, Norway, for DNA extraction. Details of the sample storage and DNA extraction procedure can be found in Schregel et al. (2012). Once unique individuals were identified, one aliquot per individual was sent to our laboratory in Umeå, Sweden, for SNP genotyping.

Single nucleotide polymorphism genotyping was performed on the Fluidigm Biomark using the SNP panel as described in Norman et al. (2013) with a slight alteration: Two of the SNPs that were found to be linked (snp163 and snp171 from Norman et al. 2013) were removed and replaced with two Y-chromosome SNPs (Bidon et al. 2015). We manually screened the genotype clusters by the Biomark software and removed any loci with ambiguous cluster affiliation from further analyses. Negative controls (i.e., water in place of DNA) were included in each run. Samples that were close to the negative control were deemed “No Calls”. Duplicates (*n* = 91) and triplicates (*n* = 10) of samples were included for the estimation of genotyping error. Allelic dropout was calculated from heterozygote loci as recommended by Broquet and Petit (2004).

Sex of each sample was determined through both the Y-chromosome and X-chromosome markers. If the sample appeared in the cluster for each Y-chromosome marker, it was recorded as a male. If the sample was a “No Call”, it was considered to be a female. Any sample outside the cluster, but not at the origin (i.e., where the negative controls are located), was invalidated. The Y-chromosome determination of sex was then validated through three X-chromosome SNPs by ensuring that any male had only one allele at each X-chromosome marker, hence appearing as a homozygote, and a female was confirmed if it had at least one heterozygote genotype on the X-chromosome. Likewise, mitochondrial haplotype for each sample was determined by allelic state for each of the four diagnostic mitochondrial markers.

### Sample locations

Our first step in determining natal dispersal distance was to estimate home range centers for each individual using fecal sample locations. As many individuals’ home ranges overlap, using the center-to-center distances will provide an estimate of even short-distance dispersers. As our sample locations are based on fecal sites, we rely on the assumption that the fecal sites are within the home range. A previous study by Bellemain et al. (2005) within the same area showed that 80% of the fecal sites were found within the home range (estimated as 95% MCP) and those that were outside the home range were within 10 km of the home range. These results suggest that the fecal sites are most likely to be representative of the home range and those that are not are likely to be close by, thereby keeping the margin of error low. For individuals with multiple samples, the estimate of home range centers should be more accurate than for those with just one. For this study, we have calculated the center points as the median center using the R package “aspace” version 3.2 (Bui et al. 2012) for individuals with two or more sample locations (*n* = 138). Those with one location were maintained as is (*n* = 275). The median center was chosen due to its insensitivity to outliers, which can be indicative of an individual leaving his/her home range temporarily.

### Pedigree reconstruction

Pedigrees were reconstructed using FRANz software version 1.9.999 (Riester et al. 2009) with maximum number of females and males (Nfmax and Nmmax) set to 663 and 516, respectively. These numbers inform the software about the estimated number of missing individuals and were calculated according to the population census estimate of 810 for the region (Kindberg and Swenson 2014) based on the sex ratio found in the samples. The empirically determined typing error of 1.538 × 10^−4^ was specified. The parentage output file was filtered for those individuals with at least one parent and a posterior probability ≥0.95. Only parentage inferences that passed these filters were used in subsequent analyses.

### Estimation of relatedness

Lynch–Ritland relatedness coefficients (Lynch and Ritland 1999) were calculated for each pair to further assess relatedness between individuals using the R package “related” version 0.8 (Pew et al. 2014). The Lynch–Ritland relatedness coefficient was chosen as it has been shown to perform better than other relatedness estimators (Thomas 2005; Csilléry et al. 2006). The reconstructed pedigrees were screened for relatedness categories as follows: PO, FS (full siblings), HS (half siblings), GG (grandparent–grandchild), and mates and plotted against the coefficient of relatedness using R (R Development Core 2013).

### Isolation by distance

To determine whether the sample scope would be large enough to capture the majority of dispersal events, we tested for isolation by distance (IBD). Pairwise Euclidean distances were calculated with the median centers for all pairs of sampled individuals using Pythagorean theorem from the coordinates based on the Swedish RT90 projection. To detect IBD, a Mantel test was run for only those individuals identified as putative parents in the pedigree reconstruction results. Euclidean distance and Lynch–Ritland relatedness coefficient matrices were input into *mantel.randtest* in the R package “adegenet” version 1.4-2 (Jombart and Ahmed 2011). Three categories were computed: (1) all pairwise putative parents; (2) female–female pairs only; and (3) male–male pairs only. Additionally, a Pearson’s product-moment correlation was calculated for these three categories as well as for pairs of the opposite sex.

### Estimation of natal dispersal distance

The pedigrees were assessed to detect possible cubs based on three factors: (1) each of the cubs has a full sibling; (2) the full siblings were in the same geographic location; and (3) this geographic location was within 1 km of their mother. Individuals identified as cubs were subsequently removed from natal dispersal distance analysis as they have not yet dispersed. Natal dispersal distances were calculated for all remaining offspring with a known mother as identified in the reconstructed pedigrees. Finally, a Wilcoxon rank-sum test was applied to female and male dispersal distances to determine whether there was a significant difference using R (R Development Core 2013).

### Biases

Spatial and logistical limitations may cause biases. As we are using noninvasively collected samples from a portion of the population that is continuous beyond the area sampled, we will inevitably miss some dispersal events and particularly long-distance events. As brown bear exhibits male-biased dispersal (Swenson et al. 1998; McLellan and Hovey 2001; Proctor et al. 2004; Støen et al. 2006), missing these long-distance dispersal events will underestimate distances for males in particular. Likewise, through noninvasive sampling alone, there is no current method to determine the age of individuals. As juveniles disperse between the ages of 2–5 years (Støen et al. 2006), there will likely be individuals accounted for that have not yet dispersed, leading to a possible underestimation of distances. Finally, deviations from true home range centers may lead to slight under- or overestimations.

## Results

### SNP genotyping

We successfully genotyped 433 individuals from the 434 uniquely identified individual DNA extracts we received at 96 SNP loci. One was unsuccessful due to probable contamination and was therefore removed from all further analyses. Within all heterozygote SNP loci (*n* = 7825) excluding the haploid SNPs (Y-chromosome and mtDNA), we identified three probable genotyping errors resulting in an error rate of 0.00038. There were 134 (0.36%) autosomal genotypes that were invalidated due to inability to resolve which cluster it belonged to. Thus, the call rate for all SNPs excluding the Y-chromosome was 0.9965. Mean minor allele frequency for autosomal SNPs was 0.37. We identified 243 females and 190 males through the Y-chromosome and X-chromosome markers. All of the individuals shared the same mitochondrial haplotype that is representative of the southern Swedish population (see Norman et al. 2013) with the exception of seven males, six of which have the haplotype common to the middle population and one to the northern population indicating possible long-distance dispersal.

### Sample locations

**Figure 2:**
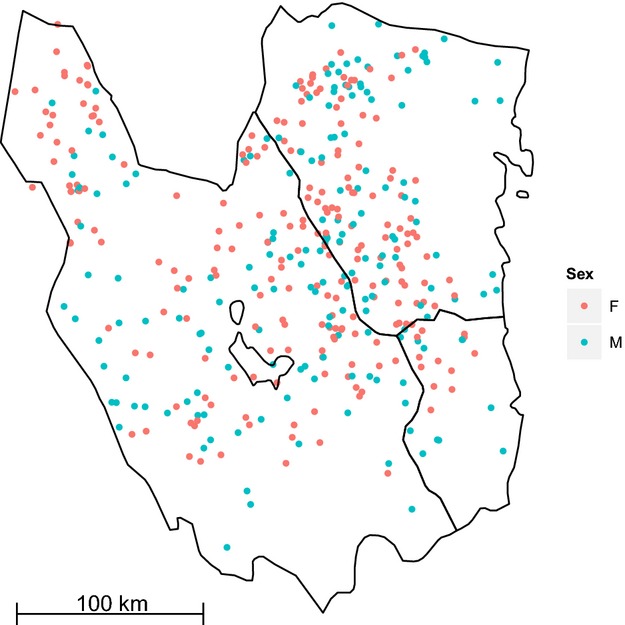
Map of Gävleborg and Dalarna counties in mid-Sweden where a large portion of the south Swedish population of brown bears occurs. Each point represents one individual (*N* = 412; missing from map *N* = 21). Where multiple samples per individual were collected, the point represents the median center of all samples.

Of the 433 genotyped individuals, we had coordinate data for 412 individuals. Mean and maximum number of samples collected per individual were 2.20 (SD: 2.59) and 19, respectively. Overall mean distance between sample sites of the same individual was 15.6 km (SD: 11.4). Table1 shows the frequency distribution of number of samples per individual. A map showing the median centers for individuals with multiple samples and single point locations for those individuals with one sample is shown in Figure2.

**Table 1:** Frequency distribution of the number of samples collected per individual

Number of samples	Number of individuals	Percentage of all individuals
1	275	66.4
2	45	10.9
3	25	6.0
4	24	5.8
5	10	2.4
6–10	25	6.0
11–15	7	1.7
>=16	3	0.7

### Pedigree reconstruction

Of the 433 individuals, FRANz identified two parents for 65 individuals, one parent for 172 individuals, and no parents for 196 individuals. From those with at least one parent identified, the posterior probability was greater than or equal to 0.95 for 82 individuals: 60 triads (both parents identified) and 22 dyads (one parent identified). The total number of unique individuals comprising these triads and dyads is 149. In total, these triads and dyads make up 28 disjoint pedigrees ranging in size from 2 to 13 individuals (mean: 5.36; SD: 3.54) and spanning two to three generations.

### Estimation of relatedness

**Figure 3:**
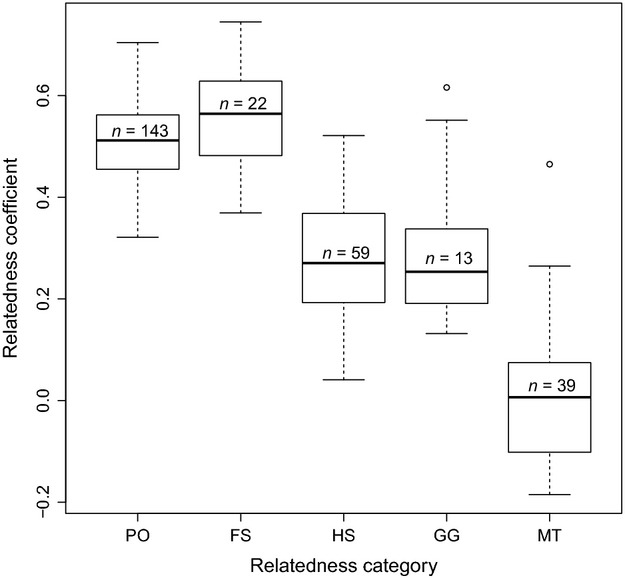
Representation of categorical relationships identified through reconstructed pedigrees and the associated coefficient of relatedness (*r*) (Lynch and Ritland 1999). PO represents parent–offspring pairs; FS represents full-sibling pairs; HS represents half-sibling pairs; GG represents grandparent–grandoffspring pairs; and MT represents mate pairs. PO and FS are first-order relatives with an expected *r*-value of 0.50. HS and GG are second-order relatives with an expected *r*-value of 0.25. Mates are expected to be unrelated with an *r*-value of 0.0. Outliers are represented by open circles and are found in both GG and MT indicating possible inbreeding events.

The Lynch–Ritland relatedness coefficient (*r*) was calculated for all pairs within the sampled individuals (*N* = 93,528 pairwise comparisons). The results were then subset for all pairs of individuals contained within the pedigrees (*N* = 11,027). Mean relatedness was −0.0023 (SD: 0.1270) and −0.0003 (SD: 0.1424) within all sampled individuals and the pedigreed individuals, respectively. Figure3 shows the categorical relationships (PO, FS, HS, GG, and MT) and their associated *r*-estimates of the pedigreed pairs (*N* = 132). These results fall into the scope of what can be expected for each relatedness category, indicating that the pedigrees and *r*-estimates are in agreement with one another. There are two outliers, one in a grandparent–grandchild (GG) pair and one in a MT (mated pair). Both appear at the upper end of the *r*-scale, which can be indicative of pairs with unusually high levels of inbreeding (in the GG pair) and mates who are closely related (in the MT pair). Both outliers are therefore retained in subsequent analyses.

### Isolation by distance

**Figure 4:**
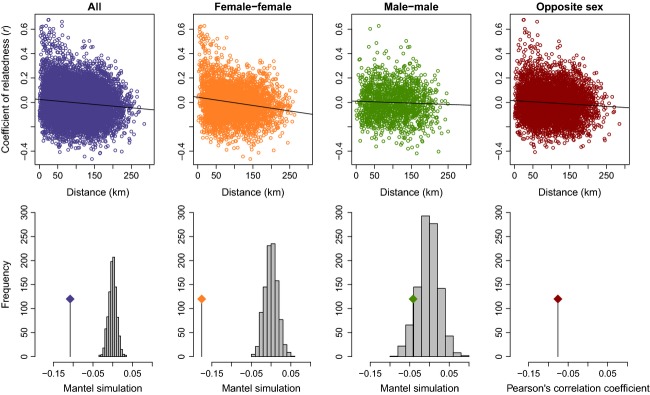
The top scatterplots have pairwise Euclidean distance on the *X*-axis and pairwise Lynch–Ritland coefficient of relatedness on the *Y*-axis and a linear regression line to indicate the overall trend for each of four categories of individuals designated as putative parents: all pairs, female–female pairs, male–male pairs, and opposite-sex pairs. The bottom graphs show the results of a Mantel test for IBD (isolation by distance) for each of the above-mentioned categories with the exception of opposite-sex pairs, which are represented with the Pearson’s correlation value. The further away the test statistic is from the simulated bars, the greater the significance of IBD.

Euclidean distance between all pairs of sampled individuals based on the median centers for those with multiple locations resulted in a mean of 100.6 km and SD of 53.3. Isolation by distance was significant for all putative parent pairs (*N* = 9870; Mantel correlation: −0.11; *P*-value < 0.001) and female–female pairs (*N* = 3655; Mantel correlation: −0.18; *P*-value < 0.001) and nonsignificant for male–male pairs (*N* = 1485; Mantel correlation: −0.042; *P*-value = 0.080) (Fig.4). Additionally, the Pearson’s correlation test (a statistic that is comparable to the Mantel test with the type of data used in this study) was applied to all categories above and additionally to pairs of the opposite sex for which a Mantel test could not be applied due to its asymmetrical nature. Pearson’s correlation for all categories is as follows: all: −0.11 (*N* = 9870); female pairs: −0.18; male pairs: −0.041; and opposite sex: −0.076 (*N* = 4730).

### Natal dispersal

From the pedigree analysis, of the 82 offspring with at least one parent identified, 71 included the mother. Of these 71 offspring, eight were identified as cubs (see Materials and Methods for identification technique) and subsequently removed from the natal dispersal distance analyses, leaving 63 mother–offspring pairs.

**Figure 5:**
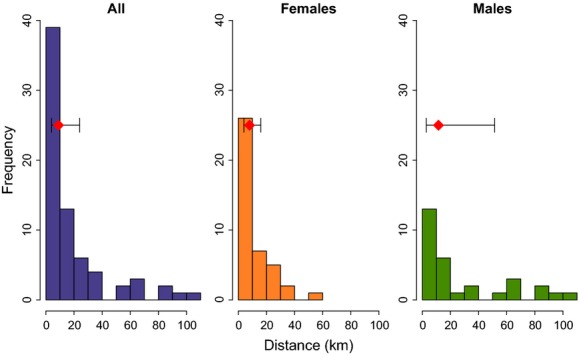
Frequency histogram showing the pedigree-based estimates of dispersal distance for all mother–offspring pairs (*n* = 63), mother–daughter pairs (*n* = 38), and mother–son pairs (*n* = 25). The red diamonds represent the median distance, and the lines extending from the diamonds show the interquartile ranges.

Natal dispersal distances ranged from 0 to 53 km (mean: 12.9; SD: 11.7 km) for females and 1 to 103 km (mean: 33.8; SD: 33.9 km) for males (Table2; Fig.5). A Wilcoxon rank-sum test (Mann–Whitney test) indicates a significant difference between female and male dispersal distances with a 0.05 significance level (Wilcoxon’s rank-sum test; W = 309; *P*-value = 0.02).

**Table 2:** Dispersal distance estimates showing the *N* (number of individuals), the median, mean, SE (standard error), and maximum distance for all individuals, females only, and males only. Results from a previous study by Støen et al. (2006) showing mean and SE of brown bear dispersal distances estimated from the same population as our study, but having used different methods, are shown in the final column

	*N*	Median	Mean ± SE	Max	*Previous estimates Mean ± SE (*N*; Max)
All offspring	63	11	21.2 ± 3.2	103	
Female offspring	38	9	12.9 ± 1.9	53	15.7 ± 2.4 (31; 90)
Male offspring	25	14	33.8 ± 6.8	103	108.3 ± 27.4 (16; 467)

* From Støen et al. (2006).

## Discussion

In this study, we estimated natal dispersal distances for brown bear using noninvasively collected samples and a set of 96 SNPs. We determined whether our sample scope would be large enough to capture the majority of dispersal events through a test for IBD. A significant result for females suggests that this is the case, whereas a nonsignificant result for males suggests that we are missing some of the long-distance dispersal events for males. Indeed, for a comparison with previous estimates from the same population where radio-collars were used, Støen et al. (2006) report similar female distances as our study, but longer male distances. Thus, while our estimates for females are likely to be representative of the true distances, the estimates for males are missing long distances. However, while male estimates are biased toward the shorter distances, we nevertheless detect a significant difference between female and male dispersal estimates with male dispersing further.

Støen et al. (2006) limited distances to those beyond the mother’s home range and, in some cases, to only those that were beyond the mean distance possibly leading to an upward bias. Contrarily, we opted to include all ranges of distances only excluding individuals that, based on their pedigrees, are highly likely to still be in the care of their mother. We chose to include short-distance dispersers as it can reveal population features that would otherwise be missed including kin and nonkin interactions as well as fine-scale details of philopatry such as sex ratio and variations in distances from the natal area. Natal dispersal is defined as the movement of progeny from the birthplace (the natal area) to the area where it reproduces (the breeding area) for various taxa (see Greenwood 1980; Broquet and Petit 2009; Matthysen 2012). For many small mammals in particular, the distance between natal and breeding areas can be measured as the distance between the population where the individual was born and the population where the individual reproduces (e.g., Centeno-Cuadros et al. 2011; Dey et al. 2013). Where dispersal is measured between discrete populations, rates and distances, once detected, are relatively easy to quantify. Contrarily, dispersal events for large mammals are often considered at the population scale where individuals disperse within a population as well as to neighboring populations as with the brown bear. At this scale, unless an individual remains in the direct vicinity of its mother, dispersal rates can be difficult to ascertain as it begs the question: What is a disperser and how is it distinguished from a nondisperser? Sometimes, arbitrary distance thresholds based on life-history parameters are used to make this distinction (Broquet and Petit 2009). However, given the definition, an individual can have dispersed very short distances if it has reproduced and is largely independent of its mother. Very short-distance dispersers can have a considerable effect on conservation issues such as inbreeding and population genetic structure (Greenwood 1980; Eiserhardt et al. 2013). We therefore opted to include all natal dispersal distances to appropriately describe dispersal patterns. It is worth noting that this does not preclude individuals from being considered philopatric.

The use of noninvasively collected samples enabled us to obtain information about the population without disturbing or interacting with the individuals in the study. While there are some limitations to using noninvasively collected samples such as a lack of demographic information and a limited sampling scope, the advantages make it worthwhile in comparison with other methods. Studying dispersal in large carnivores such as the brown bear is difficult as the animals are elusive, highly mobile, and potentially dangerous to researchers. Not only that, but they are sensitive to the mere presence of humans. A study by Ordiz et al. (2013) showed that just the scent of a human nearby affected the behavior of the brown bear for up to 2 days afterward. Other methods, such as tracking radio-collared individuals, require individuals to be captured through sedation. Capturing individuals is in itself challenging as it is expensive often requiring the use of a helicopter, ethical permits, and the presence of a veterinarian. However, the main concern is the negative consequences on the individuals captured with the worst case scenario being death (Arnemo et al. 2006). In comparison, the use of noninvasively collected samples is ideal. This is true for other large carnivores, but also for many species, large and small, which are sensitive to capture and handling or difficult to detect.

Since the advent of high-throughput sequencing, the use of SNPs in studies of wild populations has been on the rise. This study further exemplifies the advantages of SNPs over other molecular markers. For high confidence, a high sampling resolution combined with a highly informative panel of molecular markers with low error rate is recommended (Pemberton 2008). In this study, more than 50% of the population was sampled leading to a high chance of finding enough individuals within a pedigree to obtain pedigree links and to detect triads, thereby resolving the issue with directionality. Additionally, the panel of SNPs was designed to be most informative for inferring relatedness within the population under study. With a mean minor allele frequency >0.37, the cumulative power of the SNPs to distinguish between individuals is high with a probability of identity below 6 × 10^−24^. Furthermore, with one genotyping error for every 2600 loci, the chance of a false-positive relationship appearing is minimal.

## Conclusion

In this study, we have shown that it is possible to estimate natal dispersal distance in a wild population without any interaction with the individuals included in the study or any behavioral or life-history data. Despite a potential bias toward short-distance dispersers, particularly for males, the large sampling scope enabled us to detect significant male-biased dispersal and IBD in females. Two key factors contributed to this achievement. One is the high sampling resolution made possible by citizen science. It would have otherwise been challenging to obtain such a high sampling resolution in the short amount of time required. Additionally, as the citizens volunteered their time, the cost was kept low. The second key factor is that we used a highly informative SNP panel that was carefully designed for inferring relatedness in this particular population. As public databases are rapidly acquiring genetic data for wild species, the cost and time required to develop a SNP panel in other wild species will be less of a hindrance than it has been in the recent past. In addition, the bioinformatics involved in developing a SNP panel is less cumbersome than for many other applications, such as whole-genome sequencing, yet the value of it for a species of conservation concern is great.
